# Coexisting Thyroiditis and Carditis in a Patient With Lyme Disease: Looking for a Unifying Diagnosis

**DOI:** 10.1016/j.aace.2022.02.003

**Published:** 2022-02-15

**Authors:** Paria Zarghamravanbakhsh, Farzane Saeidifard, Gourg Atteya, Swetha Murthi, Ira Nash, Nicholas T. Skipitaris, Leonid Poretsky

**Affiliations:** 1 Division of Endocrinology, Northwell Health, The Donald and Barbara Zucker School of Medicine at Hofstra/Northwell, New York, New York; 2 Department of Medicine, Lenox Hill Hospital, Northwell Health, The Donald and Barbara Zucker School of Medicine at Hofstra/Northwell, New York, New York; 3 Division of Preventive Cardiology, Department of Cardiovascular Medicine, Mayo Clinic, Rochester, Minnesota; 4 Department of Cardiovascular Disease, Northwell Health, The Donald and Barbara Zucker School of Medicine at Hofstra/Northwell, New York, New York

**Keywords:** Lyme disease, thyroiditis, carditis, AV, atrioventricular, ECG, electrocardiogram, NR, normal range, T4, thyroxine, TSH, thyroid-stimulating hormone

## Abstract

**Background/Objective:**

Lyme disease, the most common vector-borne infection in the United States, causes multisystem inflammation. We describe a patient who presented with symptoms of Lyme disease, carditis, and thyroiditis.

**Case Report:**

A 53-year-old woman developed fatigue and dyspnea on exertion 1 month after returning from a trip to Delaware. Her electrocardiogram (ECG) showed first-degree atrioventricular (AV) block with a P-R interval up to 392 milliseconds, in the setting of elevated free thyroxine and undetectable thyroid-stimulating hormone levels. Lyme serology was positive. She was hospitalized and started on ceftriaxone. During the second day of hospitalization, AV block worsened to second-degree Mobitz type II but converted back to first-degree AV block after a few hours. Her 24-hour I-123 thyroid uptake and scan revealed markedly diminished I-123 uptake of 1.2%. On day 4, the P-R interval improved, and she was discharged on doxycycline for 3 weeks. P-R interval on ECG and repeated thyroid function tests were normal after finishing antibiotic treatment.

**Discussion:**

In our patient, known exposure to the vector, a classic rash on the chest, improvement in the symptoms, and normalization of thyroid function tests after antibiotic therapy support Lyme infection as a cause of carditis and painless, autoimmune thyroiditis.

**Conclusion:**

Our case highlights the importance of considering Lyme disease as a cause of painless, autoimmune thyroiditis, especially in patients with concurrent cardiovascular involvement.

**Highlights**

• Autoimmune thyroiditis can be caused by Lyme disease.
• Thyroiditis and carditis can be presenting features of Lyme disease.
• Treatment of Lyme disease presenting with Lyme carditis and thyroiditis with antibiotics leads to normalization of thyroid function tests and cardiac function.

**Clinical Relevance**

Our case highlights the importance of considering Lyme disease as a cause of subacute thyroiditis, especially in patients with concurrent cardiovascular involvement, which, to our knowledge, was reported only in 1 case previously.

## Introduction

Lyme disease, a tick-borne infection caused by the spirochete *Borrelia burgdorferi*, is the most common vector-borne infection in the United States, with an approximate incidence rate of 300,000 cases annually. Lyme disease can present in 3 stages. The early localized stage (days to weeks after the tick bite) includes skin manifestations and flu-like symptoms. The early disseminated stage (weeks to months after exposure) manifests as systemic inflammation, including cardiac involvement. The late-stage (months to years after exposure) includes neurologic manifestations.^1^ Lyme carditis has been reported in 4% to 10% of patients with Lyme disease who did not receive treatment in the early localized stage of the infection.^2^ Lyme carditis manifests mainly as conduction derangement in the atrioventricular (AV) node. Pericarditis, endocarditis, myocarditis, pericardial effusion, myocardial infarction, coronary artery aneurysm, QT-interval prolongation, and tachyarrhythmia have also been described.^2^ Unlike carditis, thyroid dysfunction coinciding with Lyme disease has rarely been reported.^3^ To our knowledge, only 1 publication reported a case of Lyme disease that presented with both carditis and thyroiditis.^3^ The definitive unifying diagnosis is complicated because of the possibility of false-positive Lyme serology in patients with thyroiditis^4^^,^^5^ and AV node block as a result of thyrotoxicosis

## Case Report

**Fig. 1:**
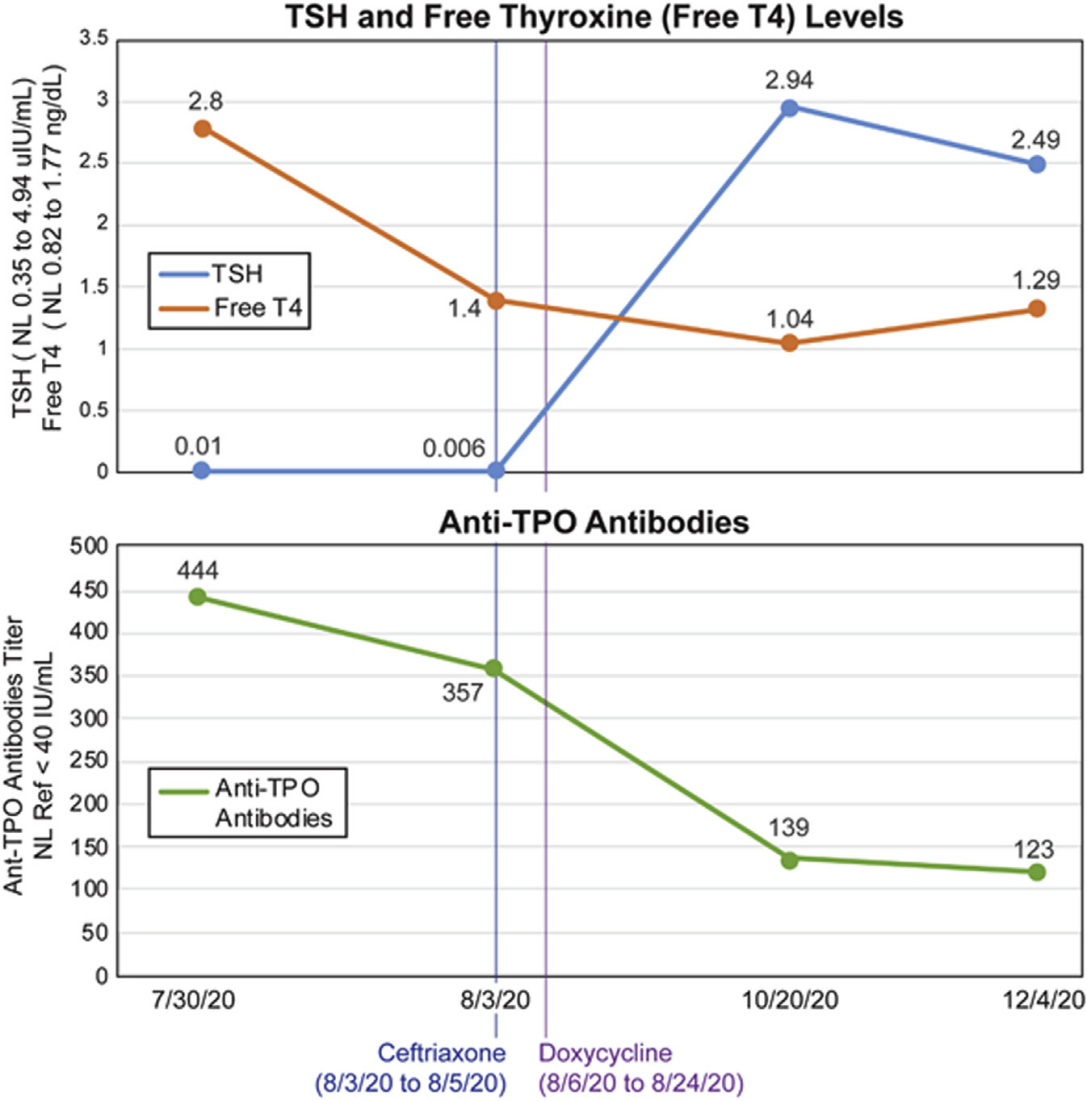
The course of thyroid function tests in a patient with Lyme thyroiditis and Lyme carditis before and after treatment with antibiotics.

**Fig. 2:**
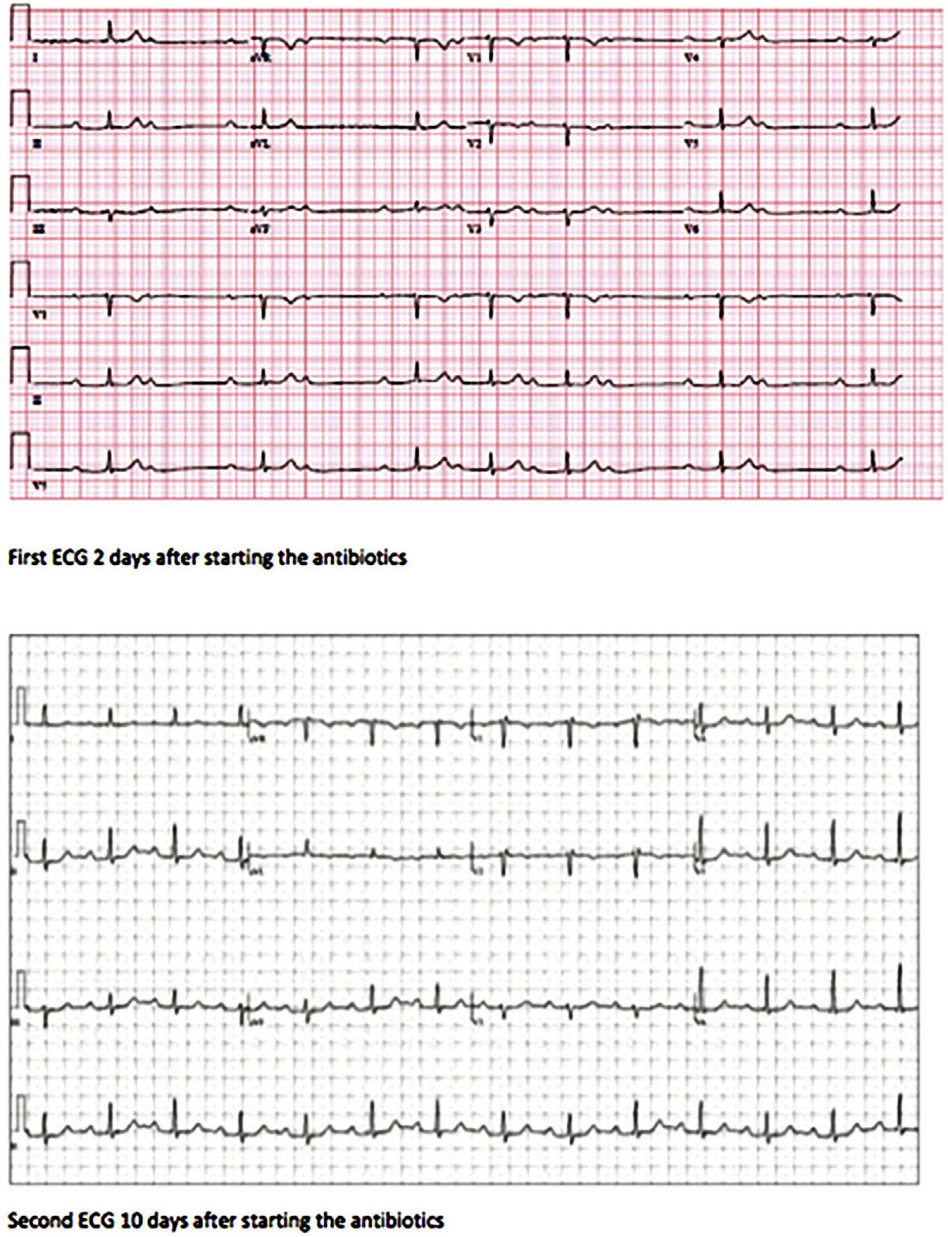
Electrocardiograms (ECGs) 2 days and then 10 days after starting antibiotics.

A 53-year-old woman with a history of subarachnoid hemorrhage and intracerebral aneurysm initially presented to an urgent care facility because of dyspnea on exertion and palpitations, occurring several weeks after her return from a 4-week stay in Delaware. While there, she had several encounters with deer while watching the sunset. Five days after her return, she developed headaches, heat intolerance, near-syncope, and a red, 5 × 5-cm rash on her chest. A few days later, she traveled for 8 days to upstate New York, where she developed dyspnea on exertion and palpitations. She presented to an urgent care facility 3 days after her return to New York City. An electrocardiogram (ECG) showed a first-degree AV block; thyroid function tests and Lyme serology were obtained. Due to the ECG findings, she was referred to a cardiologist. She denied any neck or throat pain. She also denied taking any medications or supplements, including biotin or iodine. On physical examination, blood pressure was 115/85 mm Hg, heart rate 81 beats/min, and respiratory rate 18/min. The thyroid was not enlarged or tender. No lid lag or proptosis was observed. An echocardiogram showed a moderate pericardial effusion without signs of tamponade and a mildly dilated aortic root (3.7 cm). Her free thyroxine (T4) was 2.8 ng/dL (normal range (NR), 0.70-1.48 ng/dL) and thyroid-stimulating hormone (TSH) was <0.01 mIU/L (NR, 0.35-4.94 mIU/L). Thyroid peroxidase antibodies were present at a titer of 444 IU/mL, thyroid-stimulating immunoglobulin was absent (Fig. 1). Lyme IgG/IgM test was positive. Repeat ECG showed P-R interval >300 msec (Fig. 2). Due to these findings, she was referred to the emergency department. Of note, her TSH was normal at 1.94 mIU/L (NR, 0.49-4.7 mIU/L) in another facility 4 months before her symptoms.

In the hospital, ECG showed a first-degree AV block with a P-R interval of 322 msec. Repeated TSH was 0.006 mIU/L with a total T4 of 11.78 μg/dL (NR, 4.5-11.70 μg/dL) and free T4 of 1.42 ng/dL (Fig. 1). Erythrocyte sedimentation rate was 55 mm/hr (NR, <26 mm/hr), and C-reactive protein was 0.8 mg/L (NR, 0.00-0.40 mg/L). Complete blood count, kidney function and liver function tests were within normal limits. Lyme IgG/IgM test was positive, with a titer of 8.76 units/ml. This result was confirmed with positive Lyme antibody Western Blot for IgG and IgM. With presumed Lyme carditis, she was started on ceftriaxone 2 gm/day. During the second day of hospitalization, AV block worsened to second-degree Mobitz type II (Fig. 2) but converted back to first-degree AV block after a few hours. During the third day of hospitalization, she remained in normal sinus rhythm with first-degree AV block, and the P-R interval decreased to 320 msec.

Her iodine-123 thyroid scan and 24-hour uptake showed a decrease in iodine uptake of 1.2%, consistent with thyroiditis. An echocardiogram showed normal left and right systolic and diastolic function, along with a small pericardial effusion without signs of tamponade.

After starting ceftriaxone, her P-R interval on ECG decreased to 316 msec, and symptoms improved. On day 4 of hospitalization, she was discharged on doxycycline 100 mg twice a day for 3 weeks. One week after discharge, she was asymptomatic, and ECG showed a P-R interval of 234 msec (Fig. 2).

After 8 weeks, TSH was 2.94 mIU/L with free T4 1.04 μg/dL and total T3 87 ng/dL; after 10 weeks, TSH was 2.49 mIU/L and free T4 1.29 μg/dL (Fig. 1). Her TSH was normal at 2.01 mIU/L 4 months after her admission.

## Discussion

This case report describes a 53-year-old woman who was found to have carditis and painless, autoimmune thyroiditis, likely due to Lyme infection.

The primary manifestations of Lyme carditis are non-specific and can occur 4 days to 7 months after the initial infection.^2^^,^^6^^,^^7^ Lyme carditis is a potentially fatal condition, and its early diagnosis and treatment are essential to prevent complications. Although Lyme carditis usually presents as a form of self-limited conduction derangement, it can worsen and cause complications, including higher-grade AV blocks, ventricular or supraventricular tachyarrhythmias, and sudden cardiac death.^6^^,^^8^, ^9^, ^10^

Previous studies suggested that in Lyme carditis, the deposition of neutrophils, macrophages, and lymphocytes produces an inflammatory reaction that contributes to an inflammatory cascade that ultimately causes fibrosis.^2^^,^^11^^,^^12^ Several studies have demonstrated the presence of spirochetes in the biopsies of infected patients’ hearts and large vessels.^13^^,^^14^ It is unknown whether the presence of spirochete is necessary for the continued disease or if the inflammatory cascade itself causes multisystem inflammation.

Previous reports described an association between Lyme disease and thyroid disease. Paparone^15^ described a case in which Lyme disease was superimposed on primary hypothyroidism and made the diagnosis of *Borrelia* infection challenging due to similar manifestations in both diseases. Dhliwayo et al^16^ reported a case of a 22-year old woman with Lyme disease whose thyroid function tests were consistent with transient thyrotoxicosis and decreased radioiodine uptake in the thyroid.

Any form of thyroid dysfunction, especially thyrotoxicosis, can put the patient at risk for further worsening of cardiac function. Deol et al^3^ described the association of Lyme disease with thyroiditis in a 40-year-old man who presented with night sweats and weakness. The patient also had high-grade second-degree AV block, elevated thyroid hormone levels, suppressed TSH, positive thyroperoxidase antibody, and decreased iodine uptake in the thyroid.^3^

The bioinformatics data by Benvenga et al^17^ suggested a possible explanation for thyroid involvement in patients with Lyme infection. They reported amino-acid sequence homologies between certain microbial proteins and thyroid autoantigens, suggesting that the molecular mimicry between *B. burgdorferi* and human antigens can introduce autoimmunity.

To further complicate the issue, Lyme serology can be false-positive for various reasons, including treponemal disease, systemic lupus erythematosus, rheumatoid arthritis, etc. False-positive seroreactivity to *B. burgdorferi* in a patient with thyroiditis can be caused by antibodies to self-antigens and other bacteria cross-reacting with the Lyme enzyme-linked immunosorbent assay serology.^18^^,^^19^ When a positive Lyme test is found using a highly sensitive enzyme immunoassay, a Western blot test (which is highly specific for Lyme serology) can be used to differentiate true-positive versus false-positive results.

In our patient, there was a known exposure to the vector, a classic rash, and improvement in the symptoms accompanied by normalization of thyroid function tests after treatment. These findings support a cause-effect relationship between Lyme infection, carditis and thyroiditis. As mentioned above, thyroiditis can produce false-positive Lyme serology and conduction derangements in the heart.^4^^,^^20^ For this reason, in patients with abnormal thyroid function tests and positive Lyme serology but no history of tick bite or no improvement after treatment for Lyme disease, a primary diagnosis of thyroiditis should be considered.

## Conclusion

This case report demonstrates the importance of considering Lyme disease as a unifying diagnosis in patients with concurrent carditis and thyroiditis.

## Disclosure

The authors have no multiplicity of interest to disclose.
